# Antibiotic Resistance Prevalence and Trends in Patients Infected with *Helicobacter pylori* in the Period 2013–2020: Results of the European Registry on *H. pylori* Management (Hp-EuReg)

**DOI:** 10.3390/antibiotics10091058

**Published:** 2021-09-01

**Authors:** Luis Bujanda, Olga P. Nyssen, Dino Vaira, Ilaria M. Saracino, Giulia Fiorini, Frode Lerang, Sotirios Georgopoulos, Bojan Tepes, Frederic Heluwaert, Antonio Gasbarrini, Theodore Rokkas, Dmitry Bordin, Sinead Smith, Vincent Lamy, María Caldas, Elena Resina, Raquel Muñoz, Ángel Cosme, Ignasi Puig, Francis Megraud, Colm O’Morain, Javier P. Gisbert, on behalf of the Hp-EuReg Investigators

**Affiliations:** 1 Centro de Investigación Biomédica en Red de Enfermedades Hepáticas y Digestivas (CIBERehd), Hospital Donostia/Instituto Biodonostia, Universidad del País Vasco (UPV/EHU), 20014 San Sebastián, Spain; angel.cosmejimenez@osakidetza.net; 2 Centro de Investigación Biomédica en Red de Enfermedades Hepáticas y Digestivas (CIBERehd), Hospital Universitario de La Princesa, Instituto de Investigación Sanitaria Princesa (IIS-IP), Universidad Autónoma de Madrid (UAM), 28006 Madrid, Spain; opn.aegredcap@aegastro.es (O.P.N.); m.caldas.a@gmail.com (M.C.); elenaresina93@gmail.com (E.R.); rakel_143@hotmail.com (R.M.); javier.p.gisbert@gmail.com (J.P.G.); 3 Department of Surgical and Medical Sciences, University of Bologna, 40138 Bologna, Italy; berardino.vaira@unibo.it (D.V.); saracinoilariamaria@gmail.com (I.M.S.); giulia.fiorini@fastwebnet.it (G.F.); 4 Gastroenterology Unit, Medical Department, Østfold Hospital Trust, 1714 Grålum, Norway; flerang@online.no; 5 Gastroenterology Unit, Athens Medical Paleo Faliron Hospital, 17562 Athens, Greece; p.faliro@iatriko.gr; 6 AM DC Rogaska, 3250 Rogaska Slatina, Slovenia; Bojan.tepes@siol.net; 7 Gastroenterology Unit, Centre Hospitalier Annecy Genvois, 74370 Pringy, France; fheluwaert@ch-annecygenevois.fr; 8 Medicina Interna, Fondazione Policlinico Universitario A. Gemelli IRCCS, Università Cattolica del Sacro Cuore, 00168 Roma, Italy; Antonio.gasbarrini@unicatt.it; 9 Gastroenterology Unit, Henry Dunant Hospital, 11526 Athens, Greece; sakkor@otenet.gr; 10 Gastroenterology Unit, A.S. Loginov Moscow Clinical Scientific Center, 111123 Moscow, Russia; dbordin@mail.ru; 11 Gastroenterology Unit, Department of Clinical Medicine, Trinity College Dublin, D24 NR0A Dublin, Ireland; smithsi@tcd.ie (S.S.); colmomorain@gmail.com (C.O.); 12 CHU de Charleroi, B-6000 Charleroi, Belgium; dr.vincent.lamy@gmail.com; 13 Althaia Xarxa Assistencial Universitària de Manresa and Universitat de Vic-Universitat Central de Catalunya (UVicUCC), 08242 Manresa, Spain; ignasipuig@gmail.com; 14 Unit INSERM 1053, Université de Bordeaux, 33076 Bordeaux, France; francis.megraud@chu-bordeaux.fr; 15 Gastroenterology Unit, Department of Outpatient Therapy and Family Medicine, Tver State Medical University, 170100 Tver, Russia; 16 Gastroenterology Unit, Department of Propaedeutic of Internal and Gastroenterology, A.I. Yevdokimov Moscow State University of Medicine and Dentistry, 127473 Moscow, Russia

**Keywords:** *Helicobacter pylori*, amoxicillin, clarithromycin, metronidazole, levofloxacin, tetracycline, antibiotic resistance

## Abstract

*Background***:** Bacterial antibiotic resistance changes over time depending on multiple factors; therefore, it is essential to monitor the susceptibility trends to reduce the resistance impact on the effectiveness of various treatments. *Objective***:** To conduct a time-trend analysis of *Helicobacter pylori* resistance to antibiotics in Europe. *Methods***:** The international prospective European Registry on *Helicobacter pylori* Management (Hp-EuReg) collected data on all infected adult patients diagnosed with culture and antimicrobial susceptibility testing positive results that were registered at AEG-REDCap e-CRF until December 2020. *Results*: Overall, 41,562 patients were included in the Hp-EuReg. Culture and antimicrobial susceptibility testing were performed on gastric biopsies of 3974 (9.5%) patients, of whom 2852 (7%) were naive cases included for analysis. The number of positive cultures decreased by 35% from the period 2013–2016 to 2017–2020. Concerning naïve patients, no antibiotic resistance was found in 48% of the cases. The most frequent resistances were reported against metronidazole (30%), clarithromycin (25%), and levofloxacin (20%), whereas resistances to tetracycline and amoxicillin were below 1%. Dual and triple resistances were found in 13% and 6% of the cases, respectively. A decrease (*p* < 0.001) in the metronidazole resistance rate was observed between the 2013–2016 (33%) and 2017–2020 (24%) periods. *Conclusion*: Culture and antimicrobial susceptibility testing for *Helicobacter pylori* are scarcely performed (<10%) in Europe. In naïve patients, *Helicobacter pylori* resistance to clarithromycin remained above 15% throughout the period 2013–2020 and resistance to levofloxacin, as well as dual or triple resistances, were high. A progressive decrease in metronidazole resistance was observed.

## 1. Introduction

*Helicobacter pylori* (*H. pylori*) is one of the most common bacterial infections worldwide, affecting over 40% of the population. Prevalence rates range widely among geographic regions; the highest levels are seen in developing countries, showing well-established relationships with socioeconomic status and hygiene conditions [1,2]. There are numerous situations where treatment is indicated, such as in patients with peptic ulcer disease, a family history of gastric cancer, or dyspepsia. In 1994, the International Agency for Research on Cancer declared *H. pylori* as a group 1 carcinogen for gastric cancer [3], and more recently, given its high rate of resistance to clarithromycin, it was declared by the World Health Organization (WHO) as a pathogen with high priority for the need of new antimicrobial drugs [4].

Clarithromycin resistance rate has been increasing since 1998 [5], although in a lesser extent in Europe since 2008 [6]; a subsequent reduction of success rates of clarithromycin-based triple therapy was an indirect indication of increasing resistance [7,8]. Antibiotic misuse is the primary cause of the increase in resistance. Exposure to antibiotics is very high: up to 46% of the patients for whom an eradication treatment was indicated had received macrolides (mainly clarithromycin and azithromycin) in the previous 12–14 years [9]. In this study, the exposure to other antibiotics as amoxicillin, metronidazole, and quinolones were 80%, 11%, and 4%, respectively [9]. Throughout the period 2000–2010, global macrolide and fluoroquinolone consumption increased by 19% and 64%, respectively [10,11]. These antimicrobial agents are usually prescribed for respiratory, genital, or urinary infections, and parasite infections. The highest primary resistance rate registered for metronidazole relates to the massive use of this antibiotic to treat parasitic infections or other infections for urinary tract or gastrointestinal by anaerobes [10]. Eradication of *H. pylori* infection is not achieved with exposure to (isolated) antibiotics that are being taken for other infections and leads to the development of resistance [12]. Several studies show low antibiotic resistance when no treatment with a specific antibiotic has been previously given, e.g., clarithromycin, and in this scenario, the effectiveness of triple therapy is optimal (90%) [10,13,14]. On the other hand, a correlation between antibiotic administration and resistance to clarithromycin and levofloxacin has been recently proven in Europe [6,13].

An evaluation of bacterial antibiotic susceptibility patterns is needed to improve treatment effectiveness. For optimal cure rates (>90%), selecting treatment parameters (susceptible antibiotics, high doses of proton pump inhibitors, and longer treatment durations) is essential to avoid known local resistances [8,15]. Thus, most international guidelines recommend not using clarithromycin when resistance to this antibiotic is >15% [7].

Levofloxacin is also frequently used for eradicating *H. pylori* infection, generally as a second-line treatment. Over the last years, resistances >15% have been observed in some geographical areas, which may condition the success of *H. pylori* eradication. *H. pylori* also shows high resistance rates to metronidazole, which may occasionally reach even >40% [10]. Despite this, many treatment schemes include metronidazole, either as a standard quadruple regimen or as a single-capsule formulation in combination with tetracycline and bismuth (Pylera^®^, Allergan, Dublin, Ireland) [16]. It has been suggested that nitroimidazole resistance may be partially overcame in vivo when used in quadruple therapies.

Antibiotic resistance is not steady and may fluctuate over the years and between different regions [6]. Knowing the trend of drug resistance patterns is key to design strategies to decrease the development of resistance and help clinicians improving treatment guidelines.

The aim of this study was to evaluate the resistance rates of *H. pylori* to the different antibiotics in Europe as well as its evolution over the years, stratified by country and line of *H. pylori* eradication treatment.

## 2. Results

### 2.1. Characteristics of the Participants

**Figure 1:**
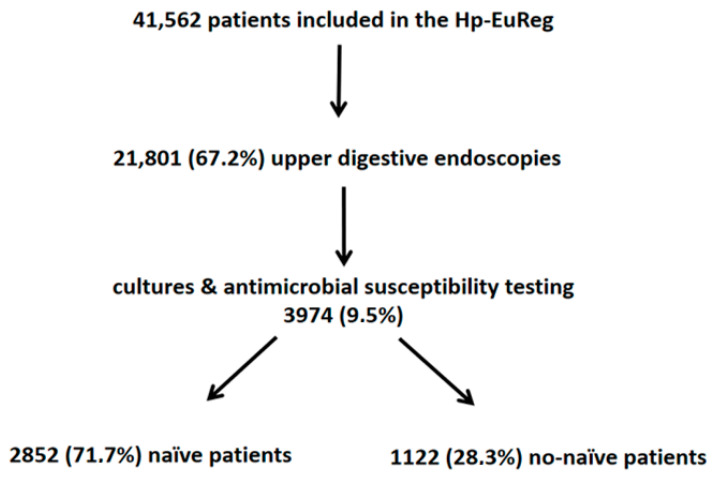
Flow chart of patients included in the study.

The Hp-EuReg included 41,562 patients by December 2020. Culture was performed on 3974 (9.5%) individuals, of whom 2852 (71.7%) were naïve and 1122 non-naïve patients (Figure 1). The mean age was 51 (±15) years, and 2463 (62%) were women; 3615 (91%) of the participants were of Caucasian origin. Diagnostic indications for *H. pylori* testing and culture were as follows: 2893 (72.7%) functional dyspepsia, 237 (6%) duodenal ulcers, 102 (2.6%) gastric ulcers, 78 (2%) uninvestigated dyspepsia, and 664 (16.7%) patients with other conditions.

The geographical distribution of cases was as follows: 2,360 (59%) in Italy; 454 (11.4%) in Spain; 368 (9.3%) in Norway; 248 (6.2%) in Greece; 211 (5.3%) in Slovenia; 110 (2.8%) in Israel; 45 (1.1%) in France; and 40 (1%) in Ireland. The number of *H. pylori* isolates by country and year is reported in Supplementary Table S1.

### 2.2. H. pylori Antibiotic Resistance in Naïve Patients

No antibiotic resistance was reported in 1365 (48%) of the cases. Most commonly detected resistances were related to metronidazole (852, 30%), clarithromycin (701, 25%), and levofloxacin (561, 20%). Amoxicillin and tetracycline resistances were infrequent: 11 (0.4%) and five (0.2%) cases, respectively.

Dual clarithromycin/metronidazole resistance was found in 368 (13%) cases, and triple clarithromycin/metronidazole/levofloxacin resistance in 172 (6%).

### 2.3. Evolution of Antibiotic Resistance from 2013 to 2020 in Naïve Patients

**Figure 2:**
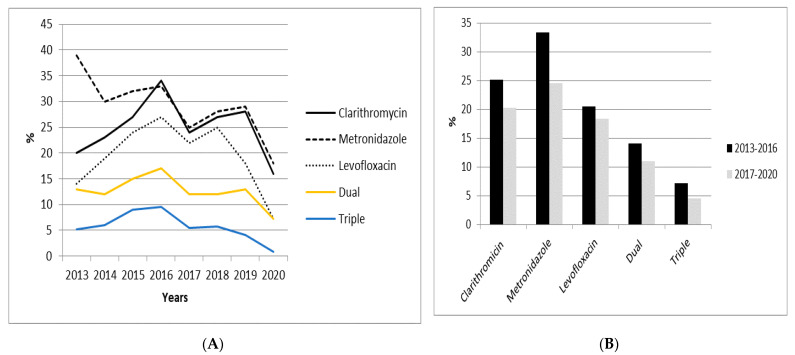
Evolution of *Helicobacter pylori* antibiotic resistance in naïve patients over the past eight years (**A**) and according to study periods: 2013–2016 and 2017–2020 (**B**).

Table 1 details the antibiotic resistance in naïve patients over the study’s eight-year period (from 2013 to 2020). The average resistance rate to clarithromycin was 25% (16–34%), with a maximum peak in 2016, reaching 34% (Figure 2A). Levofloxacin resistance remained above 15% during the study, except in 2013 and particularly in 2020, when resistances were 14% and 7.3%, respectively.

A significant (*p* < 0.001) decrease in the metronidazole resistance rate was observed between 2013 (39%) and 2020 (18%). Resistances to amoxicillin and tetracycline remained below 1% throughout the study period.

Dual clarithromycin and metronidazole resistance was greater than 10% during most of the study period, except in 2020, when a resistance rate of 7.3% was reported. However, in 2015 and 2016, dual resistance was reported to be over 15%. Triple clarithromycin, metronidazole, and levofloxacin resistance was greater than 5%, except in 2019 (4.1%) and 2020 (0.9%).

The analysis of each of the antibiotics’ primary resistance rates when comparing both study periods (2013–2016 and 2017–2020) showed a decreasing trend for each of the antibiotics. Such decrease was significantly greater for metronidazole, ranging from 33.3% in the first period to 24.5% in the second one (*p* < 0.05) (Table 2).

### 2.4. H. pylori Resistance in Naïve Patients per Country and Geographical Area

The level of *H. pylori* resistance in southern European countries was higher than in northern Europe (Norway) (56% vs. 31.5%, respectively; *p* < 0.005) (Table 3). Resistance in southern Europe was greater than 20% for clarithromycin and levofloxacin, as opposed to Norway with rates below 10% for the same antibiotics (Table 3). A similar trend was seen for dual and triple resistances in southern Europe (15% and 7.5%, respectively) versus northern Europe (3.5% and 0.3%, respectively). Supplementary Table S2 depicts the different antibiotics’ bacterial resistances according to the country of the naïve patients.

### 2.5. H. pylori Antibiotic Resistance in Non-Naïve Patients

Resistance was found in over 80% of the non-naïve patients. Clarithromycin resistance following a first eradication treatment attempt was greater than 60%. Levofloxacin resistance after a first eradication treatment was 28%, increasing to over 45% when the patient had received more than two eradication treatments (Supplementary Table S3). Both resistance to amoxicillin and tetracycline remained below than 2% in all treatment lines.

Dual and triple resistances after the failure of the first eradication treatment were found in 43% and 19% of patients, respectively. These resistances progressively increased after the failure of four eradication treatments, reaching rates up to 63% and 39%, respectively. 

When comparing the rates of antibiotic resistance between naïve and non-naïve patients who had undergone one eradication, a bigger proportion of patients with resistance to any of the evaluated antibiotics was observed in non-naïve patients, except for amoxicillin and tetracycline that remained low and stable in both groups (Table 4).

## 3. Discussion

Antibiotic resistance is a worldwide public health concern that involves different bacteria. In 2017, the WHO classified *H. pylori* as a *high priority* pathogen due to the increased number of cases of clarithromycin resistance [4]. In this study, we observe that the number of cultures performed in order to monitor *H. pylori* resistance was low (˂10%), and the overall resistance to clarithromycin and levofloxacin over the past eight years was high, especially in southern European countries.

To establish the best treatments, i.e., to obtain a maximum efficacy and a minimum of adverse events, it is necessary to know which antibiotic resistance has actually developed in each patient. Ideally, antimicrobial susceptibility testing (AST) should be performed for all diagnosed patients, as is the case for patients with urinary tract infections. Evidence suggests that such a strategy is more effective than empiric therapy [17,18,19,20,21]. Moreover, it has been reported to be cost-effective, particularly in regions with high resistance rates [17,18,19,20,21]. However, in standard clinical practice, *H. pylori* AST is not always possible as this procedure involves invasive testing such as gastroscopy. In general, few centers perform culture and AST systematically as part of the routine clinical practice, such practice being frequently linked to specific research projects [6,22,23,24]. Another factor that may explain the scarcity of data is that *H. pylori* isolation requires special pre-analytical conditions and attention in the laboratory; the methods must be carefully followed, which explains why a positive result may be achieved in less than 70% of the cases in some laboratories [23,24], and in some centers, *H. pylori* isolation has decreased over the last years [23]. For instance, in a study performed in Spain in 2004, it was shown that *H. pylori* isolation was achieved in 64% of the cases, whilst in 2016 this proportion decreased to 41% [23]. In order to improve these results, other procedures may be used, such as the string test, which allows avoiding endoscopy [25]. However, commercially available molecular methods, especially for macrolides, can be used to allow both detection of *H. pylori* and its resistance to clarithromycin from gastric biopsies with better sensitivity than culture [26]. These methods can even be applied to stool samples when endoscopy is not necessary. The limit is the low amount of *H. pylori* DNA in stools, but DNA extraction methods are improving and provide satisfactory results [27].

In this study, bacterial clarithromycin and levofloxacin resistance was found in 25% and 20% of naïve patients, respectively, with both above the 15% threshold generally used to determine high bacterial resistance. In this scenario, most guidelines do not recommend the use of triple therapy with clarithromycin or levofloxacin but suggest a quadruple therapy with or without bismuth. After a first failed treatment, resistance increases remarkably and reaches over 66% and 28% to clarithromycin and levofloxacin, respectively, after a fourth treatment attempt. In 2020, a decrease in the resistance rate was observed for the aforementioned two antibiotics [6]. In 2020, a 20% decrease in the number of cultures was observed. The number of cultures/year in naive patients during the 2013–2019 period was 373, while in 2020, it dropped to 298 (−20%) (Supplementary Table S4). This decrease can be explained, on the one hand, by the decrease in the number of cultures done over the years, and on the other hand, by the COVID-19 pandemic that in 2020 significantly affected Europe, especially Spain and Italy. In 2020, due to the COVID-19 pandemic, non-urgent digestive endoscopy was stopped for several months in most European hospitals, paralleling a decrease in consultations for digestive diseases. It remains to be determined whether this decreasing trend will continue in the following years.

On the contrary, no significant increase in levofloxacin resistance was detected over the study period, contrarily to what has been reported by several studies performed in Asia; such is the case in Korea, where an increase in levofloxacin resistance ranged from 44% in 2013 and 2014 to 62% in 2017 and 2018 [28,29]. This may have occurred due to the use of fluoroquinolones for the treatment of respiratory or urinary tract infections or to the development of cross-resistance with other fluoroquinolones. Mutations in DNA gyrase subunit A (*gyr*A) genes play a critical role in *H. pylori* fluoroquinolone resistance. Other quinolone antibiotics, such as sitafloxacin, may overcome the resistance of *H. pylori* strains harboring *gyr*A mutations and may be an alternative in the upcoming years [28]. 

Metronidazole resistance in naïve patients has been found to be lower (30%) than reported elsewhere. Other studies found resistance in similar patients between 50 and 80%, and generally over 34% of patients [10,12,29]. A bacterial resistance rate below 30% in naïve patients treated with quadruple treatments that include metronidazole may possibly allow optimal effectiveness (over 90%). The great difference in resistance in other countries may also be due to the use of this antibiotic for certain parasitic infections [30]. Conversely, amoxicillin and tetracycline resistances were very low in our study, below 1%. These data are quite different from those reported in Asian areas, where the amoxicillin resistance rate varied from 6% to 8% and the tetracycline resistance rate from 4% to 16% [29]. Overall, the rate of amoxicillin resistance in Asian regions is higher, as well as in other countries, such as the United States of America [22].

Another hurdle in the eradication treatments is the multiple antibiotic resistances and secondary resistances in non-naïve patients. As previously mentioned, dual and triple resistances in naïve patients are found in 13% and 6% of the cases, respectively. However, in non-naïve individuals, dual resistance rates may reach 43% and 19% for triple resistance. Some studies confirmed these high dual and triple resistance rates in other countries, such as Italy [31,32].

Resistance may notably vary among countries and within geographical regions in the same country. In our study, marked differences were observed between northern and southern European countries. These variations have also been reported in recent studies [6], with resistances rates to clarithromycin and levofloxacin of 28% and 23.5%, respectively, in the southern region, and 7% and 2.5%, respectively, in northern countries. Thus, monitoring the resistance prevalence in the different countries and regions of the same country is key to determine which treatment may be better tailored to each local need.

Our study has some relevant limitations. Firstly, the low proportion of cases in which AST was performed (9.5%) and also the low number of cultures performed in many countries does not allow a precise estimation of the prevalence of the different bacterial resistance in large scale geographical areas. Secondly, most results were obtained from southern European countries, where higher resistance rates have been reported [6]. Thirdly, the evaluation of resistance to rifampicin, which reports for rifabutin, the antibiotic used, were not included in this study. Finally, there were no available data on previous consumption of antibiotics in participating countries (low or high) as a determining factor of antibiotic resistance.

Strengths of this study include the international multicenter design, the ample range of years evaluated, and the fact that data reflected the daily routine in clinical practice among European gastroenterologists and not only tertiary hospitals (highly specialized).

In summary, the main conclusion of the present study is that the number of AST performed in Europe is low. *H. pylori* resistance is generally high for clarithromycin and levofloxacin, particularly in southern European countries. However, a progressive decrease in metronidazole resistance was observed. Dual and triple antibiotic resistances are also high, with a noticeable increase in non-naïve patients.

## 4. Materials and Methods

### 4.1. European Registry on Helicobacter pylori Management

The European Registry on *H. pylori* Management (Hp-EuReg) is an international multicenter prospective non-interventional registry with a collection of information on *H. pylori* infection management since 2013, which was promoted by the European Helicobacter and Microbiota Study Group (www.helicobacter.org, accessed on 1 February 2021) [33,34].

The ethics committee of La Princesa University Hospital (Madrid, Spain), which acted as the reference institutional review board, approved the protocol of the Hp-EuReg [34]. The study protocol conforms to the ethical guidelines of the 1975 Declaration of Helsinki as reflected in a prior approval by the institution’s human research committee. The study was classified by the Spanish Drug and Health Product Agency and registered at ClinicalTrials.gov under the code NCT02328131. Written informed consent was obtained from all participants.

Criteria for country selection, national coordinators, and gastroenterologist recruiting investigators, as well as the list of variables and outcomes, are detailed in the published protocol [33,34]. Eradication confirmation tests had to be available.

Data were recorded in an electronic case report form (e-CRF), collected and managed using the web-based application designed to support data capture for research studies (REDCap) [35] and hosted at the *Asociación Española de Gastroenterología* (AEG; www.aegastro.es, accessed on 1 February 2021), a non-profit scientific and medical society focused on gastroenterology research [34]. Overall, 31 European countries with over 300 recruiters were selected.

The objective of the current study was to assess the prevalence of antibiotic resistance in *H. pylori* strains in Europe as part of the Hp-EuReg study.

### 4.2. Selection Criteria

Adult patients (>18 years of age) with an *H. pylori*-positive result for culture and AST, recruited between January 2013 and December 2020, were included in the analysis. Cases without an antibiogram were excluded. 

### 4.3. Data Management and Analysis

Data extraction was performed in February 2021 and subjected to monitoring (at least 10% of the existing records per country and centre) and a quality check to ensure coherence and data reliability.

Primary antibiotic resistance was defined as bacterial resistance to one or more of the antibiotics tested (amoxicillin, clarithromycin, nitroimidazole, levofloxacin, or tetracycline) in *H. pylori* treatment-naïve patients. Secondary antibiotic resistance was determined in patients who failed eradication (non-naïve individuals). In the latter case, it was defined according to the number of eradication treatments used.

Naïve patients were defined as subjects who had never been treated for *H. pylori* and non-naïve those who had previously undergone treatment (one or more eradication treatment options). This information was obtained by a clinical questionnaire and also, in most cases, by prescription records.

Indications for *H. pylori* testing were duodenal ulcer, gastric ulcer, uninvestigated or functional dyspepsia, among other conditions. Other indications included a family history of gastric cancer, premalignant gastric lesions such as atrophic chronic gastritis and intestinal metaplasia, anemia of unknown origin, erosive gastroduodenitis, gastric lymphoma, and idiopathic thrombocytopenia. 

For *H. pylori* isolation, gastric biopsy specimens were obtained from the antrum and/or body of the stomach during endoscopic examinations. Cultures were performed on selective plates under microaerobic conditions. AST was performed with E-test strips in most of the centers; the provider was bioMérieux, Mary-L’Etoile, France. Antimicrobial resistance was determined according to the guidelines and criteria of the European Committee of Antibiotic Susceptibility Testing (EUCAST Clinical Breakpoint Tables V.9) (http://eucast.org/clincialbreakpoints/, accessed on 1 February 2021). Dual resistance was defined when there was resistance to clarithromycin and metronidazole. Triple resistance was defined when there was resistance to clarithromycin, metronidazole, and levofloxacin.

From the countries included in the study, only those with more than 40 cases for which culture testing had been performed were selected for the analysis. Data were stratified by naïve and non-naïve patients as well as by geographic areas.

To assess the prevalence of antibiotic resistance in Europe, two regions were established by their geographic location and the number of cultures performed: southern Europe (Italy, Spain, and Greece) and northern Europe (Norway). 

Two four-year periods were established to better determine the trend in the resistances: 2013–2016 and 2017–2020.

### 4.4. Statistical Analyses

The prevalence of antibiotic resistance was presented as the ratio of the number (and percentage) of the positive cultures for a given antibiotic and the total number of patients where culture and AST had been performed. Continuous variables were shown as arithmetic means and standard deviations (SD), and qualitative variables as percentages and corresponding 95% confidence intervals (95% CI). The Chi-square test was used to compare categorical variables. *p*-Values ˂ 0.05 were considered statistically significant. Time trend analyses were performed.

## Figures and Tables

**Table 1:** *Helicobacter pylori* antimicrobial resistance trends between 2013 and 2020 in naïve patients.

Resistance	Years—Number of Patients (%)								Total
	2013	2014	2015	2016	2017	2018	2019	2020	
**No resistance**	209 (49%)	260 (50%)	245 (49%)	120 (44%)	188 (55%)	135 (48%)	130 (48%)	78 (36%)	1365 (48%)
**Clarithromycin**	85 (20%)	119 (23%)	136 (27%)	91 (34%)	83 (24%)	76 (27%)	76 (28%)	35 (16%)	701 (25%)
**Metronidazole**	165 (39%)	155 (30%)	162 (32%)	90 (33%)	84 (25%)	78 (28%)	77 (29%)	40 (18%)	852 (30%)
**Levofloxacin**	58 (14%)	100 (19%)	121 (24%)	73 (27%)	75 (22%)	69 (25%)	48 (18%)	16 (7%)	561 (20%)
**Amoxicillin**	6 (1%)	0 (0%)	0 (0%)	0 (0%)	4 (1.2%)	1 (0.4%)	0 (0%)	0 (0%)	11 (0,4%)
**Tetracycline**	2 (0.5%)	1 (0.2%)	0 (0%)	1 (0.4%)	0 (0%)	0 (0%)	1 (0.4%)	0 (0%)	5 (0,2%)
**Dual (C+M)**	56 (13%)	64 (12%)	77 (15%)	45 (17%)	41 (12%)	34 (12%)	35 (13%)	16 (7%)	368 (13%)
**Triple (C+M+L)**	22 (5%)	31 (6%)	45 (9%)	26 (10%)	19 (5.5%)	19 (6%)	11 (4%)	2 (0.9%)	172 (6%)

C—clarithromycin; M—metronidazole; L—levofloxacin.

**Table 2:** *Helicobacter pylori* antibiotic resistance trends in naïve patients over the two study periods (2013–2016 and 2017–2020).

Resistance	2013–2016 Period N (%)	2017–2020 Period N (%)	*p*-Value
No resistance	834 (49%)	531 (47%)	0.4
Clarithromycin	431 (25%)	230 (20%)	0.3
Metronidazole	572 (33%)	279 (24.5%)	0.02
Levofloxacin	352 (20.5%)	208 (18%)	0.4
Amoxicillin	6 (0.3%)	5 (0.4%)	0.5
Tetracycline	4 (0.2%)	1 (0.08%)	0.4
Dual (C+M)	242 (14%)	126 (11%)	0.3
Triple (C+M+L)	124 (7.2%)	51 (4.5%)	0.4

N—number of patients analyzed; C—clarithromycin; M—metronidazole; L—levofloxacin.

**Table 3:** *Helicobacter pylori* antibiotic resistance according to European geographical areas: southern Europe (Italy, Spain, and Greece) and northern Europe (Norway), in naïve patients.

Resistance	Southern Europe N (%)	Northern Europe N (%)	*p*-Value
No resistance	996 (44%)	218 (68.5%)	0.03
Clarithromycin	631 (28%)	22 (7%)	0.001
Metronidazole	687 (30.5%)	83 (26%)	0.4
Levofloxacin	530 (23.5%)	8 (2.5%)	0.001
Amoxicillin	5 (0.2%)	0 (0%)	0.3
Tetracycline	3 (0.1%)	1 (0.3%)	0.4
Dual (C+M)	333 (15%)	11 (3.5%)	0.02
Triple (C+M+L)	168 (7.5%)	1 (0.3%)	0.02

N—number of patients analyzed; C—clarithromycin; M—metronidazole; L—levofloxacin.

**Table 4:** *Helicobacter pylori* resistance against different antibiotics tested in naïve and non-naïve patients.

Resistance	Naïve N (%)	Non-Naïve N (%)	*p*-Value
No resistance	1365 (48%)	93 (16%)	0.001
Clarithromycin	701 (25%)	380 (66%)	0.01
Metronidazole	852 (30%)	309 (54%)	0.02
Levofloxacin	561 (20%)	164 (28%)	0.03
Amoxicillin	11 (0.4%)	4 (0.7%)	0.3
Tetracycline	5 (0.2%)	0 (0%)	0.4
Dual (C+M)	368 (13%)	248 (43%)	0.02
Triple (C+M+L)	172 (6%)	109 (19%)	0.01

N—number of patients analyzed; C—clarithromycin; M—metronidazole; L—levofloxacin.

## Supplementary Materials

The following are available online at https://www.mdpi.com/article/10.3390/antibiotics10091058/s1, Table S1: The number of Helicobacter pylori isolates by country and year, Table S2: Distribution of primary antimicrobial resistances in Europe (by country with more than 20 cases included), Table S3: Overall antimicrobial resistances by line of treatment, Table S4: Helicobacter pylori antibiotic resistance trends in naïve patients over the two study periods (2013–2016, 2017–2019 and 2020)
