# Supplementary material for: Antibiotic Resistance Prevalence and Trends in Patients Infected with Helicobacter pylori in the Period 2013–2020: Results of the European Registry on H. pylori Management (Hp-EuReg)
Source: Antibiotics (Basel). 2021 Sep 1;10(9):1058. doi: 10.3390/antibiotics10091058 (PMC8471667; doi:10.3390/antibiotics10091058)
Supplement: Supplementary file 1 [file antibiotics-10-01058-s001.zip › Supplementary File.pdf]

Supplementary tables

Table S1. The number of *Helicobacter pylori* isolates by country and year.

	2013	2014	2015	2016	2017	2018	2019	2020
**Italy**	214	346	379	242	318	354	275	206
**Spain**	15	128	143	99	30	15	20	4
**Norway**	141	138	78	9	2	0	0	0
**Greece**	41	45	34	30	25	23	34	16
**Slovenia**	74	34	2	1	0	11	53	33
**Israel**	0	0	21	23	12	8	6	34
**Russia**	15	29	2	1	0	6	0	0
**France**	16	4	6	4	15	0	0	0
**Ireland**	27	10	2	0	0	1	0	0
**Total**	543	734	667	409	402	418	388	293

Table S2. Distribution of primary antimicrobial resistances in Europe (by country with more than 20 cases included).

N (%)	Italy	Spain	Norway	Greece	Slovenia	Russia	France	Ireland
**No resistance**	692 (42%)	212 (54%)	218 (67%)	92 (41%)	90 (61%)	17 (35%)	13 (65%)	13 (41%)
**Clarithromycin**	489 (30%)	57 (15%)	22 (7%)	85 (38%)	20 (14%)	10 (24%)	2 (10%)	10 (31%)
**Metronidazole**	517 (32%)	104 (27%)	83 (26%)	66 (29%)	41 (28%)	14 (29%)	1 (5%)	15 (47%)
**Levofloxacin**	444 (27%)	68 (17%)	8 (2.5%)	18 (8%)	5 (3%)	13 (27%)	3 (15%)	1 (3%)
**Amoxicillin**	5 (0.3%)	0 (0%)	0 (0%)	0 (0%)	2 (1%)	3 (6%)	0 (0%)	1 (3%)
**Tetracycline**	1 (0.1%)	2 (0.5%)	1 (0.3%)	0 (0%)	1 (0.7%)	0 (0%)	0 (0%)	0 (0%)
**Dual (C+M)**	286 (18%)	20 (5%)	11 (3.5%)	27 (12%)	12 (8%)	1 (2%)	0 (0%)	7 (22%)
**Triple (C+M+L)**	157 (10%)	9 (2%)	1 (0.3%)	2 (0.9%)	0 (0%)	1 (2%)	0 (0%)	1 (3%)

N: number of patients analysed; C = clarithromycin; M = metronidazole; L = levofloxacin.

Table S3. Overall antimicrobial resistances by line of treatment.

N (%)	First-line (naïve)	Second-line	Third-line	Fourth-line	Fifth-line	Sixth-line
**No resistance**	1,365 (48%)	93 (16%)	38 (11%)	12 (9%)	5 (12%)	1 (6%)
**Clarithromycin**	701 (25%)	380 (66%)	264 (74%)	99 (78%)	33 (81%)	11 (69%)
**Metronidazole**	852 (30%)	309 (54%)	237 (66%)	83 (65%)	29 (71%)	10 (63%)
**Levofloxacin**	561 (20%)	164 (28%)	164 (46%)	58 (46%)	20 (49%)	6 (38%)
**Amoxicillin**	11 (0.4%)	4 (0.7%)	6 (1.7%)	2 (1.6%)	0 (0%)	0 (0%)
**Tetracycline**	5 (0.2%)	0 (0%)	2 (0.6%)	2 (1.6%)	0 (0%)	0 (0%)
**Dual (C+M)**	368 (13%)	248 (43%)	200 (56%)	72 (57%)	26 (63%)	8 (50%)
**Triple (C+M+L)**	172 (6%)	109 (19%)	122 (34%)	45 (35%)	16 (39%)	6 (38%)

N: number of patients analysed; C = clarithromycin; M = metronidazole; L = levofloxacin.

Table S4. *Helicobacter pylori* antibiotic resistance trends in naïve patients over the two study periods (2013-2016, 2017-2019 and 2020).

Resistance	2013-2016 period N (%)	2017-2019 period N (%)	2020 year
**No resistance**	834 (49%)	453 (51%)	78 (36%)
**Clarithromycin**	431 (25%)	235 (26%)	35 (16%)
**Metronidazole**	572 (33%)	239 (27%)	40 (18%)
**Levofloxacin**	352 (20.5%)	192 (21.5%)	16 (7%)
**Amoxicillin**	6 (0.3%)	5 (0.4%)	0 (0.4%)
**Tetracycline**	4 (0.2%)	1 (0.1%)	0 (0.1%)
**Dual (C+M)**	242 (14%)	110 (12%)	16 (7%)
**Triple (C+M+L)**	124 (7.2%)	49 (5.5%)	2 (0.9%)
**Total cultures**	1716	895	219

N: number of patients analyzed; C: clarithromycin; M: metronidazole; L: levofloxacin.

ONLINE SUPPLEMENTARY MATERIAL

Supplementary file 1. Hp-EuReg Investigators

Jen Hinojosa Agencia Sanitaria Costa del Sol Marbella SPAIN Acquired data, critically reviewed the manuscript draft, and approved the submitted manuscript.

Inmaculada Santaella Agencia Sanitaria Costa del Sol Marbella SPAIN Acquired data, critically reviewed the manuscript draft, and approved the submitted manuscript.

Nuria Fernandez Moreno Agencia Sanitaria Costa del Sol Marbella SPAIN Acquired data, critically reviewed the manuscript draft, and approved the submitted manuscript.

Ilaria Maria Saracino University of Bologna ITALY Acquired data, critically reviewed the manuscript draft, and approved the submitted manuscript.
Horacio Alonso Galán Hospital de Donisti San Sebastian SPAIN Acquired data, critically reviewed the manuscript draft, and approved the submitted manuscript.
Almudena Durán Hospital de la Princesa Madrid SPAIN Acquired data, critically reviewed the manuscript draft, and approved the submitted manuscript.
Jennifer Fernandez Pacheco Hospital de la Princesa Madrid SPAIN Acquired data, critically reviewed the manuscript draft, and approved the submitted manuscript.
Miroslav Vujasinovic Slovenj Gradec General Hospital Slovenj Gradec SLOVENIA Acquired data, critically reviewed the manuscript draft, and approved the submitted manuscript.
Rinaldo Pellicano Molinette Hospital Turin ITALY Acquired data, critically reviewed the manuscript draft, and approved the submitted manuscript.
Zdenko Kikec General Hospital Slovenj Gradec Slovenj Gradec SLOVENIA Acquired data, critically reviewed the manuscript draft, and approved the submitted manuscript.
Pedro-Luis Gonzalez Cordero Hospital San Pedro de Alcantara Caceres SPAIN Acquired data, critically reviewed the manuscript draft, and approved the submitted manuscript.
Maia Donday Hospital de la Princesa, CIBERehd, Instituto de Salud Carlos III Madrid SPAIN Monitored and interpreted the data, critically reviewed and approved the final manuscript
Angel Lanas Hospital Clinico Universitario Lozano Blesa Zaragoza SPAIN Acquired data, critically reviewed the manuscript draft, and approved the submitted manuscript.
Polona Lampic Diagnosticni center Bled Bled SLOVENIA Acquired data, critically reviewed the manuscript draft, and approved the submitted manuscript.
Vid Leban Diagnosticni center Bled Bled SLOVENIA Acquired data, critically reviewed the manuscript draft, and approved the submitted manuscript.
Aleksander Gruncic Krajnc Diagnosticni center Bled d.o.o. Bled SLOVENIA Acquired data, critically reviewed the manuscript draft, and approved the submitted manuscript.
Galyna Dmytrivna Fadieienko Ukrainian Academy of Medical Sciences Kharkiv UKRANIA Acquired data, critically reviewed the manuscript draft, and approved the submitted manuscript.
Lorena Lee Instituto Fundación Teófilo Hernando Madrid SPAIN Monitored and interpreted the data, critically reviewed and approved the final manuscript
Irene V Barbado Instituto Fundación Teófilo Hernando Madrid SPAIN Monitor and interpret the data, critically reviewed and approved the final manuscript
Alfredo José Lucendo Hospital General de Tomelloso Tomelloso SPAIN Acquired data, critically reviewed the manuscript draft, and approved the submitted manuscript.
Jesus Barrio RIo Hortega Hospital Valladolid SPAIN Acquired data, critically reviewed the manuscript draft, and approved the submitted manuscript.
Tatiana Alekseevna Ilchishina SM-clinic Saint-Petersburg RUSSIA Acquired data, critically reviewed the manuscript draft, and approved the submitted manuscript.
Irina Voynovan Moscow Scientific Clinical Center Moscow RUSSIA Acquired tdata, critically reviewed the manuscript draft, and approved the submitted manuscript.
Luis Ignacio Fernández-Salazar Hospital Clínico Universitario Valladolid SPAIN Acquired data, critically reviewed the manuscript draft, and approved the submitted manuscript.
Jose María Huguet Hospital General Universitario de Valencia Valencia SPAIN Acquired data, critically reviewed the manuscript draft, and approved the submitted manuscript.
Pilar Canelles Hospital General Universitario de Valencia Valencia SPAIN Acquired data, critically reviewed the manuscript draft, and approved the submitted manuscript.

Aiman Silkanovna Sarsenbaeva Gastroenterological center Chelyabinsk RUSSIA Acquired data, critically reviewed the manuscript draft, and approved the submitted manuscript.
Ines Modolell Consorci Sanitari Terrassa Terrassa SPAIN Acquired data, critically reviewed the manuscript draft, and approved the submitted manuscript.
Pedro Almela Hospital General Universitario de Castellon Castellon SPAIN Acquired data, critically reviewed the manuscript draft, and approved the submitted manuscript.
Marina Roldán Lafuente Hospital General Universitario de Castellón Castellón de la Plana SPAIN Acquired data, critically reviewed the manuscript draft, and approved the submitted manuscript.
Josep Maria Botargues Hospital Universitari de Bellvitge L'Hospitalet de Llobregat SPAIN Acquired data, critically reviewed the manuscript draft, and approved the submitted manuscript.
Miguel Areia Portuguese Oncology Institute Coimbra PORTUGAL Acquired data, critically reviewed the manuscript draft, and approved the submitted manuscript.
Luís Elvas Portuguese Oncology Institute Coimbra PORTUGAL Acquired data, critically reviewed the manuscript draft, and approved the submitted manuscript.
Susana Isabel Alves Portuguese Oncology Institute Coimbra PORTUGAL Acquired data, critically reviewed the manuscript draft, and approved the submitted manuscript.
Daniel Brito Portuguese Oncology Institute Coimbra PORTUGAL Acquired data, critically reviewed the manuscript draft, and approved the submitted manuscript.
Ana Teresa Cadime Portuguese Oncology Institute Coimbra PORTUGAL Acquired data, critically reviewed the manuscript draft, and approved the submitted manuscript.
Sandra Lúcia Madeira Saraiva Oncologycal Hospital of Coimbra Coimbra PORTUGAL Acquired data, critically reviewed the manuscript draft, and approved the submitted manuscript.
Charalampos Tzathas Tzaneio General Hospital Pireaus GREECE Acquired data, critically reviewed the manuscript draft, and approved the submitted manuscript.
Vassiliki Ntouli Tzaneio General Hospital Pireaus GREECE Acquired data, critically reviewed the manuscript draft, and approved the submitted manuscript.
Alicia C Marin Hospital de la Princesa, CIBERehd, Instituto de Salud Carlos III Madrid SPAIN Monitore and interpretd the data, critically reviewed the manuscript draft, and approved the submitted manuscript.
Cem Simsek Hacettepe University Ankara TURKEY Acquired data and approved the submitted manuscript
Gerardo Nardone University Federico II Naples ITALY Acquired data, critically reviewed the manuscript draft, and approved the submitted manuscript.
Alba Rocco University Federico II Naples ITALY Acquired data, critically reviewed the manuscript draft, and approved the submitted manuscript.
Juan Antonio Ortuño Hospital Universitari i Politècnic La Fe Valencia SPAIN Acquired data, critically reviewed the manuscript draft, and approved the submitted manuscript.
Tommaso Di Maira University Hospital La Fe Valencia SPAIN Acquired data, critically reviewed the manuscript draft, and approved the submitted manuscript.
Sotirios D. Georgopoulos Athens Medical Center, Paleo Faliron General Hospital Athens GREECE Acquired data, critically reviewed the manuscript draft, and approved the submitted manuscript.
Stephan Brackmann Lovisenberg Diakonale Hospital Oslo NORWAY Acquired data, critically reviewed the manuscript draft, and approved the submitted manuscript.
Vendel Kristensen Lovisenberg Diakonale Hospital Oslo NORWAY Acquired data, critically reviewed the manuscript draft, and approved the submitted manuscript.

Blas Jose Gomez-Rodriguez Hospital Quiron Sagrado Corazon Seville SPAIN Acquired data, critically reviewed the manuscript draft, and approved the submitted manuscript.
Perminder Singh Phull Aberdeen Royal Infirmary Aberdeen U.K. Acquired data, critically reviewed the manuscript draft, and approved the submitted manuscript.
Sergey Alekseyevich Alekseenko Far Eastern State Medical University Khabarovsk RUSSIA Acquired data, critically reviewed the manuscript draft, and approved the submitted manuscript.
Monica Perona Hospital Quirón Marbella SPAIN Acquired data, critically reviewed the manuscript draft, and approved the submitted manuscript.
Rustam Abdulkhakov Kazan State Medical University Kazan RUSSIA Acquired data, critically reviewed the manuscript draft, and approved the submitted manuscript.
Deirdre McNamara Adelaide & Meath Hospital Tallaght Dublin IRELAND Acquired data, critically reviewed the manuscript draft, and approved the submitted manuscript.
Sinead M. Smith Trinity College Dublin Dublin IRELAND Acquired data, critically reviewed the manuscript draft, and approved the submitted manuscript.
Denise Elizabeth Brennan Trinity College Dublin Dublin IRELAND Acquired data, critically reviewed the manuscript draft, and approved the submitted manuscript.
Marina Fedorovna Osipenko Novosibirsk State Medical University Novosibirsk RUSSIA Acquired data, critically reviewed the manuscript draft, and approved the submitted manuscript.
Cristobal de la Coba Hospital de Cabueñes Gijon SPAIN Acquired data, critically reviewed the manuscripts draft, and approved the submitted manuscript.
Pilar Varela Hospital de Cabueñes Gijon SPAIN Acquired data, critically reviewed the manuscript draft, and approved the submitted manuscript.
Maria Anatolyevna Livzan Omsk state medical academy Omsk RUSSIA Acquired data, critically reviewed the manuscript draft, and approved the submitted manuscript.
Oleg V. Zaytsev First Clinical Medical Center Kovrov RUSSIA Acquired data, critically reviewed the manuscript draft, and approved the submitted manuscript.
Vladislav Vladimirovich Tsukanov Federal State Budgetary Institution, Scientific research institute of medical problems of the North Siberian branch under the Russian Academy of Medical Sciences (FSBI 'SRIMPN' SB RAMS) Krasnoyarsk RUSSIA Acquired data, critically reviewed the manuscript draft, and approved the submitted manuscript.
Alexander Viktorovich Vasyutin Federal State Budgetary Scientific Institution Scientific research institute of medical problems of the North Krasnoyarsk RUSSIA Acquired data, critically reviewed the manuscripts draft, and approved the submitted manuscript.
Olga Sergeevna Amelchugova Federal State Budgetary Scientific Institution Scientific research institute of medical problems of the North Krasnoyarsk RUSSIA Acquired data, critically reviewed the manuscript draft, and approved the submitted manuscript.
Spiros Michopoulus Alexandra Hospital Athens GREECE Acquired data, critically reviewed the manuscript draft, and approved the submitted manuscript.
Sergey Gennadievich Burkov Outpatient clinic Moscow RUSSIA Acquired data, critically reviewed the manuscript draft, and approved the submitted manuscript.
Dan Dumitrascu Medical Department 2, University of Medicine and Pharmacy Iuliu Hatieganu Cluj Napoca ROMANIA Acquired data, critically reviewed the manuscript draft, and approved the submitted manuscript.
Bogdan Ianosi Medical Department 2, University of Medicine and Pharmacy Iuliu Hatieganu Cluj Napoca ROMANIA Acquired data, critically reviewed the manuscript draft, and approved the submitted manuscript.

Ingrid Prytz Berset Alesund Hospital Alesund NORWAY Acquired data, critically reviewed the manuscript draft, and approved the submitted manuscript.
Rafael Ruiz -Zorrilla Lopez Hospital de Sierrallana Torrelavega SPAIN Acquired data, critically reviewed the manuscript draft, and approved the submitted manuscript.
Charo Antón Clinico de Valencia Valencia SPAIN Acquired data, critically reviewed the manuscript draft, and approved the submitted manuscript.
Anne Courillon-Mallet Centre Hospitalier Intercommunal (CIHV) Villeneuve St Georges FRANCE Acquired data, critically reviewed the manuscript draft, and approved the submitted manuscript.
Natasa Brglez Jurecic Splosna bolnisnica Trbovlje Trbovlje SLOVENIA Acquired data, critically reviewed the manuscript draft, and approved the submitted manuscript.
Maja Denkovski: Interni oddelek, Diagnostic Centre, 4260 Bled, Slovenia
(maja.denkovski@gmail.com)
Judith Gomez-Camarero Hospital Universitario de Burgos Burgos SPAIN Acquired data, critically reviewed the manuscript draft, and approved the submitted manuscript.
Manuel Jimenez-Moreno Hospital Universitario de Burgos Burgos SPAIN Acquired data, critically reviewed the manuscript draft, and approved the submitted manuscript.
Ahmet Uygun GATA Hospital Ankara TURKEY Acquired data, critically reviewed the manuscript draft, and approved the submitted manuscript.
Ian Leonard Phillip Beales Norfolk and Norwich University Hospital Norwich
U.K. Acquired data, critically reviewed the manuscript draft, and approved the submitted manuscript.
Alain Huerta-Madrigal Hospital Universitario Madrid Sanchinarro Madrid SPAIN Acquired the data, critically reviewed the manuscript draft, and approved the submitted manuscript.
Javier Alcedo Hospital de Barbastro Huesca SPAIN Acquired the data, critically reviewed the manuscript draft, and approved the submitted manuscript.
Mercè Barenys Hospital de Viladecans Viladecans SPAIN Acquired data, critically reviewed the manuscript draft, and approved the submitted manuscript.
Francesco Franceschi Catholic University of Rome Rome ITALY Acquired data, critically reviewed the manuscript draft, and approved the submitted manuscript.
Jean-Charles Delchier Henri Mondor Hospital Créteil FRANCE Acquired data, critically reviewed the manuscript draft, and approved the submitted manuscript.
Liliana Silvia Pozzati Mérida Hospital Mérida SPAIN Acquired data, critically reviewed the manuscript draft, and approved the submitted manuscript.
Monika Augustyn Wojskowy Szpital Kliniczny Cracow POLAND Acquired data, critically reviewed the manuscript draft, and approved the submitted manuscript.
Maja Seruga Hospital Murska Sobota Slovenia SLOVENIA Acquired data, critically reviewed the manuscript draft, and approved the submitted manuscript.
Miriam Hiestand Hospital of Chur Chur SWITZERLAND Acquired data, critically reviewed the manuscript draft, and approved the submitted manuscript.
Patric Mosler Hospital of Chur Chur SWITZERLAND Acquired data, critically reviewed the manuscript draft, and approved the submitted manuscript.
Zaza Beniashvili Rabin Medical Center, Hospital Ha Sharon Petah Tikva ISRAEL Acquired data, critically reviewed the manuscript draft, and approved the submitted manuscript.
Yaron Niv: Rabin Medical Center, Tel Aviv University, 49100 Petah Tikva, Israel (nivyaron80@gmail.com)

Hubert Louis Hôpital Erasme Brussels BELGIUM Acquired data, critically reviewed the manuscript draft, and approved the submitted manuscript.
Ramon Pajares Hospital Universitario Infanta Sofía San Sebastian de los Reyes SPAIN Acquired data, critically reviewed the manuscript draft, and approved the submitted manuscript.
Igor Bakulin: North-western State Medical University, 191015 St Petersburg, RUSSIA (igbakulin@yandex.ru)
Natalia Valerievna Zakharova North-western State Medical University named after I.I.Mechnikov St. Petersburg RUSSIA Acquired data, critically reviewed the manuscript draft, and approved the submitted manuscript.
Natalia Nikolaevna Dekhnich Institute of Antimicrobial Chemotherapy Smolensk RUSSIA Acquired data, critically reviewed the manuscript draft, and approved the submitted manuscript.
Victor Asparuhov Kamburov Lyulin Hospital Sofia BULGARIA Acquired data, critically reviewed the manuscript draft, and approved the submitted manuscript.
Maria Pina Dore Clinica Medica, Dipartimento di Scienze Mediche, Chirurgiche e Sperimentali, Università di Sassari Sassari ITALY Acquired data, critically reviewed the manuscript draft, and approved the submitted manuscript.
Lorena Sancho Del val Hospital Rey Juan Carlos Madrid SPAIN Acquired data, critically reviewed the manuscript draft, and approved the submitted manuscript.
Noelia Alcaide Hospital Clínico Universitario Valladolid SPAIN Acquired the data, critically reviewed the manuscript draft, and approved the submitted manuscript.
Oscar Núñez Hospital Universitario Sanitas La Moraleja Madrid SPAIN Acquired data, critically reviewed the manuscript draft, and approved the submitted manuscript.
Katrine Dvergsnes Sørlandet Sykehus HF Kristiansand NORWAY Acquired data, critically reviewed the manuscript draft, and approved the submitted manuscript.
Peter Malfertheiner Otto-von-Guericke University Hospital Magdeburg GERMANY Acquired data, critically reviewed the manuscript draft, and approved the submitted manuscript.
Ana Campillo Hospital Reina Sofia Tudela SPAIN Acquired data, critically reviewed the manuscript draft, and approved the submitted manuscript.
Miguel Fernandez-Bermejo Clinica San Francisco Caceres SPAIN Acquired data, critically reviewed the manuscript draft, and approved the submitted manuscript.
Manuel Domínguez-Cajal Hospital San Jorge Huesca SPAIN Acquired data, critically reviewed the manuscript draft, and approved the submitted manuscript.
José Luis Domínguez Jiménez Alto Guadalquivir Hospital Andújar SPAIN Acquired data, critically reviewed the manuscript draft, and approved the submitted manuscript.
Alicia Algaba Hospital Universitario de Fuenlabrada Madrid SPAIN Acquired data, critically reviewed the manuscript draft, and approved the submitted manuscript.
Fernando Bermejo Hospital Universitario de Fuenlabrada Madrid SPAIN Acquired data, critically reviewed the manuscript draft, and approved the submitted manuscript.
Borislav Vladimirov University hospotal Queen Ioanna-Isul Sofia BULGARIA Acquired data, critically reviewed the manuscript draft, and approved the submitted manuscript.
László Czakó University of Szeged Szeged HUNGARY Acquired data, critically reviewed the manuscript draft, and approved the submitted manuscript.
Teresa Angueira Hospital General de Tomelloso Ciudad Real SPAIN Acquired data, critically reviewed the manuscript draft, and approved the submitted manuscript.
Eduardo Iyo Hospital Comarcal de Inca Mallorca SPAIN Acquired data, critically reviewed the manuscript draft, and approved the submitted manuscript.

Ekaterina Yuryevna Plotnikova Kemerovo State Medical Academy Kuzbass Regional Hepatology Center Kemerovo RUSSIA Acquired data, critically reviewed the manuscript draft, and approved the submitted manuscript.
Larissa Tarasova Chuvash State University named after I.N. Ulyanov, the chair of faculty therapy Cheboksary RUSSIA Acquired data, critically reviewed the manuscript draft, and approved the submitted manuscript.
Ludmila Grigorieva Chuvash State University named after I.N. Ulyanov, the chair of faculty therapy Cheboksary RUSSIA Acquired data, critically reviewed the manuscript draft, and approved the submitted manuscript.
Judith Millastre Hospital de Barbastro Barbastro SPAIN Acquired data, critically reviewed the manuscript draft, and approved the submitted manuscript.
Bruno Richard-Molard Clinique Bordeaux Nord Bordeaux FRANCE Acquired data, critically reviewed the manuscript draft, and approved the submitted manuscript.
Aldis Pukitis Paul Stradins Clinical University Hospital Riga LATVIA Acquired data, critically reviewed the manuscript draft, and approved the submitted manuscript.
Valeriy Kryvy Crimean State Medical University named after S.I.Georgievskyi Simferopol UKRANIA Acquired data, critically reviewed the manuscript draft, and approved the submitted manuscript.
Roald Torp Sykehuset Innlandet, Hamar Hamar NORWAY Acquired data, critically reviewed the manuscript draft, and approved the submitted manuscript.
Albert Tomàs Consorci Sanitari del Garraf Sant Pere de Ribes SPAIN Acquired data, critically reviewed the manuscript draft, and approved the submitted manuscript.
Edurne Amorena Complejo Hospitalario de Navarra Pamplona SPAIN Acquired data, critically reviewed the manuscript draft, and approved the submitted manuscript.
Fermin Estremera Complejo Hospitalario de Navarra Pamplona SPAIN Acquired data, critically reviewed the manuscript draft, and approved the submitted manuscript.
Rossen Nikolov University Hospital St. Ivan Rilski Sofia BULGARIA Acquired data, critically reviewed the manuscript draft, and approved the submitted manuscript.
Asghar Quasim Beacon Hospital Dublin IRELAND Acquired data, critically reviewed the manuscript draft, and approved the submitted manuscript.
Yury Aleksandrovich Kucheryavyy Moscow State University of Medicine and Dentistry named after A.I. Evdokimov Moscow RUSSIA Acquired data, critically reviewed the manuscript draft, and approved the submitted manuscript.
Natalia Baryshnikova Fisrt St-Petersburg Pavlov State Medical University St- Petersburg RUSSIA Acquired data, critically reviewed the manuscript draft, and approved the submitted manuscript.
Xavier Calvet Corporació Sanitària Universitària Parc Taulí. CIBERehd, Instituto de Salud Carlos III Departament de Medicina. Universitat Autònoma de Barcelona Sabadell SPAIN Acquired data, critically reviewed the manuscript draft, and approved the submitted manuscript.
Ariadna Figuerola Corporació Sanitària Universitària Parc Taulí. CIBERehd, Instituto de Salud Carlos III Departament de Medicina. Universitat Autònoma de Barcelona Sabadell SPAIN Acquired data, critically reviewed the manuscript draft, and approved the submitted manuscript.
Marco Romano Università degli Studi della Campania "Luigi Vanvitelli" Napoli ITALY Acquired the data, critically reviewed the manuscript draft, and approved the submitted manuscript.
Antonietta Gerarda Gravina Università degli Studi della Campania "Luigi Vanvitelli" Napoli ITALY Acquired the data, critically reviewed the manuscript draft, and approved the submitted manuscript.

Oscar Núñez "Hospital Uni. Sanitas La Moraleja Madrid SPAIN Acquired data, critically reviewed the manuscript draft, and approved the submitted manuscript.
Fazia Mana UZ Brussel Jette BELGIUM Acquired the data, critically reviewed the manuscript draft, and approved the submitted manuscript.
Pilar Sánchez-Pobre Hospital Clinico San Carlos Madrid SPAIN Acquired data, critically reviewed the manuscript draft, and approved the submitted manuscript.
Zoya Spassova University Hospital St. Ivan Rilski Sofia BULGARIA Acquired data, critically reviewed the manuscript draft, and approved the submitted manuscript.
Jesús M González-Santiago Hospital Clínico Universitario. Instituto de Investigación Biomédica de Salamanca (IBSAL). Salamanca SPAIN Acquired data, critically reviewed the manuscript draft, and approved the submitted manuscript.
Ricardo Marcos-Pinto 1. Centro Hospitalar do Porto 2. Institute of Biomedical Sciences Abel Salazar, University of Porto 3. CINTESIS, University of Porto Porto PORTUGAL Acquired data, critically reviewed the manuscript draft, and approved the submitted manuscript.
F Wolfhagen Albert Schweitzer Ziekenhuis Dordrecht THE NETHERLANDS Acquired data, critically reviewed the manuscript draft, and approved the submitted manuscript.
Svetlana Cui Pauls Stradins Clinical University Hospital Riga LATVIA Acquired data, critically reviewed the manuscript draft, and approved the submitted manuscript.
Ivonne Leeuwenburgh Sint Franciscus Gasthuis Rotterdam THE NETHERLANDS Acquired ata, critically reviewed the manuscript draft, and approved the submitted manuscript.
Driffa Moussata Lyon Sud Hospital Pierre Benite FRANCE Acquired data, critically reviewed the manuscript draft, and approved the submitted manuscript.
Adi Lahat-zok Chaim Sheba medical center and Sackler School of Medicine, Tel Aviv University ISRAEL Acquired data, critically reviewed the manuscript draft, and approved the submitted manuscript.
Sergii Hryhorovych Melashchenko Vinnitsa National Medical University Vinnytsia UKRANIA Acquired data, critically reviewed the manuscript draft, and approved the submitted manuscript.
Rasmus Goll University Hospital of North Norway Tromso NORWAY Acquired data, critically reviewed the manuscript draft, and approved the submitted manuscript.
Tatyana Vasilyevna Zhestkova Ryazan State Medical University Ryazan RUSSIA Acquired data, critically reviewed the manuscript draft, and approved the submitted manuscript.
Juris Pokrotnieks Pauls Stradins Clinical University Hospital Riga LATVIA Acquired data, critically reviewed the manuscript draft, and approved the submitted manuscript.
Philippe Émile Houcke Hôpital Claude Huriez (CHRU) Lille FRANCE Acquired data, critically reviewed the manuscript draft, and approved the submitted manuscript.
Nadiya Byelyayeva Donetsk National Medical University Donetsk UKRANIA Acquired data, critically reviewed the manuscript draft, and approved the submitted manuscript.
Marta Lozano Lanagran Hospital Quuiron Malaga SPAIN Acquired data, critically reviewed the manuscript draft, and approved the submitted manuscript.
Mette Wildner-Christensen Odense University Hospital (OUH) Svendborg DENMARK Acquired data, critically reviewed the manuscript draft, and approved the submitted manuscript.
Bengt Odman Soder Hospital Stockholm SWEDEN Acquired data, critically reviewed the manuscript draft, and approved the submitted manuscript.
Yana Valerieva University Hospital Tsaritsa Yoanna-ISUL Sofia BULGARIA Acquired data, critically reviewed the manuscript draft, and approved the submitted manuscript.

Alenka Forte MC Heliks, d.o.o. Trbovlje SLOVENIA Acquired data, critically reviewed the manuscript draft, and approved the submitted manuscript.
Antonio Cuadrado Lavin Hospital Universitario Marqués de Valdecilla Santander SPAIN Acquired data, critically reviewed the manuscript draft, and approved the submitted manuscript.
Patrice Pienkowski Hospital Center de Montauban Montauban FRANCE Acquired data, critically reviewed the manuscript draft, and approved the submitted manuscript.
Ilze Kikuste University of Latvia, Digestive diseases centre GASTRO Riga LATVIA Acquired data, critically reviewed the manuscript draft, and approved the submitted manuscript.
Dag Arne Lihaug Hoff Aalesund Hospital Aalesund NORWAY Acquired data, critically reviewed the manuscript draft, and approved the submitted manuscript.
Jane Moeller Hansen Odense University Hospital Odense DENMARK Acquired data, critically reviewed the manuscript draft, and approved the submitted manuscript.
Konrads Funka Digestive Diseases Centre GASTRO Riga LATVIA Acquired data, critically reviewed the manuscript draft, and approved the submitted manuscript.
Alla Kononova Department of General Practice Tver RUSSIA Acquired data, critically reviewed the manuscript draft, and approved the submitted manuscript.
Sergey Kolbasnikov The Head of Department of General Practice Tver RUSSIA Acquired data, critically reviewed the manuscript draft, and approved the submitted manuscript.
Michael Selgrad Otto-von Guericke University Magdeburg GERMANY Acquired data, critically reviewed the manuscript draft, and approved the submitted manuscript.
Jolanta Sumskiene Lithuanian University of Health Sciences Hospital Lithuania LITHUANIA Acquired data, critically reviewed the manuscript draft, and approved the submitted manuscript.
Jonathan Hirsch Meir Medical Center Kfar Saba ISRAEL Acquired data, critically reviewed the manuscript draft, and approved the submitted manuscript.
Francisco Javier Zozaya Larequi Hospital Universitario Donostia San Sebastián SPAIN Acquired data, critically reviewed the manuscript draft, and approved the submitted manuscript.
Alain C. Burette Chirec clinique de la Basilique and clinique Edith Cavell Brussels BELGIUM Acquired data, critically reviewed the manuscript draft, and approved the submitted manuscript.
Nora Dancs Petz-Aladar Teaching Hospital Gyor HUNGARY Acquired data, critically reviewed the manuscript draft, and approved the submitted manuscript.
Janne Rajala Herttoniemi Hospital Helsinky FINLAND Acquired data, critically reviewed the manuscript draft, and approved the submitted manuscript.
Christina Reimer Hvidovre Hospital Hvidovre DENMARK Acquired data, critically reviewed the manuscript draft, and approved the submitted manuscript.
Diogo Libanio Gastroenterology Department Instituto Portugues de Oncologia do Porto (IPO-Porto) Porto PORTUGAL Acquired data, critically reviewed the manuscript draft, and approved the submitted manuscript.
Pedro Pimentel-Nunes Gastroenterology Department Instituto Portugues de Oncologia do Porto (IPO-Porto) Porto PORTUGAL Acquired the data, critically reviewed the manuscript draft, and approved the submitted manuscript.
Ivailo Evstatiev University Hospital St. Ekaterina Sofia BULGARIA Acquired data, critically reviewed the manuscript draft, and approved the submitted manuscript.
Juozas Kupcinskas Lithuanian University of Health Sciences Kaunas LITHUANIA Acquired data, critically reviewed the manuscript draft, and approved the submitted manuscript.
Mikhail Butov State Budgetary Educational Institution of Higher Professional Education Ryazan State I.P. Pavlov Medical University of the Ministry of Public Health of Russian Federation (SB?IHPE

RSMU of Ministry of Public Health of Russian Federation) Department of Propaedeutics of Internal Diseases Ryazan RUSSIA Acquired data, critically reviewed the manuscript draft, and approved the submitted manuscript.
Peter Mensink Medisch Spectrum Twente Enschede THE NETHERLANDS Acquired data, critically reviewed the manuscript draft, and approved the submitted manuscript.
T Tang IJsselland Ziekenhuis Capelle aan de Ijssel THE NETHERLANDS Acquired data, critically reviewed the manuscript draft, and approved the submitted manuscript.
Andrey Yurevich Baranovsky North-Western State Medical University named after I.I.Mechnicov Saint-Petersburg RUSSIA Acquired data, critically reviewed the manuscript draft, and approved the submitted manuscript.
Natalya Marchenko North-Western State Medical University named after I.I. Mechnikov Saint-Patarsburg RUSSIA Acquired data, critically reviewed the manuscript draft, and approved the submitted manuscript.
Boris Bastens CHC Liege Liege BELGIUM Acquired data, critically reviewed the manuscript draft, and approved the submitted manuscript.
Lyudmila Mateva "University Hospital St. Ivan Rilski Sofia BULGARIA Acquired data, critically reviewed the manuscript draft, and approved the submitted manuscript.
Dominique Lamarque Hôpital Ambroise-Paré Boulogne-Billancourt (Paris) FRANCE Acquired data, critically reviewed the manuscript draft, and approved the submitted manuscript.
Leonardo Henry Eusebi Department of Medical and Surgical Sciences (DIMEC), University of Bologna, Gastroenterology and Endoscopy Unit, Policlinico S.Orsola-Malpighi Bologna ITALY Acquired the data, critically reviewed the manuscript draft, and approved the submitted manuscript.
Mario Ribeiro FMUP/IPATIMUP Porto PORTUGAL Acquired data, critically reviewed the manuscript draft, and approved the submitted manuscript.
M ter Borg Maxima Medisch Centrum Eindhoven THE NETHERLANDS Acquired data, critically reviewed the manuscript draft, and approved the submitted manuscript.
Alexander C. Ford Leeds Teaching Hospitals Trust Leeds U.K. Acquired data, critically reviewed the manuscript draft, and approved the submitted manuscript.
Enrique Medina Consorcio Hospital General de Valencia Valencia SPAIN Acquired data, critically reviewed the manuscript draft, and approved the submitted manuscript.
Manuel Rodriguez-Tellez Virgen Macarena University Hospital Sevilla SPAIN Acquired the data, critically reviewed the manuscript draft, and approved the submitted manuscript.
Francisco José Rancel Medina Hospital Virgen del Puerto Plasencia SPAIN Acquired the data, critically reviewed the manuscript draft, and approved the submitted manuscript.
Elisa Martin Hospital Virgen del Puerto Plasencia SPAIN Acquired data, critically reviewed the manuscript draft, and approved the submitted manuscript.
Carolina Torres Gonzalez Hospital de la Ribera Alcira (Valencia) SPAIN Acquired data, critically reviewed the manuscript draft, and approved the submitted manuscript.
Lissa Maria Franco Hospital de Cabueñes Oviedo SPAIN Acquired data, critically reviewed the manuscript draft, and approved the submitted manuscript.
